# Cytoplasmic Cargo Receptor p62 Inhibits Avibirnavirus Replication by Mediating Autophagic Degradation of Viral Protein VP2

**DOI:** 10.1128/JVI.01255-20

**Published:** 2020-11-23

**Authors:** Yahui Li, Boli Hu, Gang Ji, Yina Zhang, Chenyang Xu, Jing Lei, Chan Ding, Jiyong Zhou

**Affiliations:** a Institute of Immunology and College of Veterinary Medicine, Nanjing Agricultural University, Nanjing, China; b MOA Key Laboratory of Animal Virology, Center for Veterinary Sciences, Zhejiang University, Hangzhou, China; c Shanghai Veterinary Research Institute, CAAS, Shanghai, China; University of Kentucky College of Medicine

**Keywords:** avibirnavirus, VP2, autophagy, cargo receptor, p62

## Abstract

Selective autophagy regulates the degradation of cytoplasmic cargos, such as damaged organelles, invading pathogens, and aggregated proteins. Furthermore, autophagy is capable of degrading avibirnavirus, but the mechanism responsible for this process is unclear. Here, we show that autophagy cargo receptor p62 regulates the degradation of the avibirnavirus capsid protein VP2. Binding of p62 to VP2 enhances autophagic induction and promotes autophagic degradation of viral protein VP2. Further study showed that the interaction of p62 with viral protein VP2 is dependent on ubiquitination at the K411 site of VP2 and the ubiquitin-associated domain of p62. Mutation analysis showed that the K411R mutation of viral protein VP2 prohibits its p62-mediated degradation. Consistent with this finding, p62 lacking the ubiquitin-associated domain or the LC3-interacting region no longer promoted the degradation of VP2. Virus production revealed that the knockout of p62 but not the overexpression of p62 promotes the replication of avibirnavirus. Collectively, our findings suggest that p62 mediates selective autophagic degradation of avibirnavirus protein VP2 in a ubiquitin-dependent manner and is an inhibitor of avibirnavirus replication.

**IMPORTANCE** Avibirnavirus causes severe immunosuppression and mortality in young chickens. VP2, the capsid protein of avibirnavirus, is responsible for virus assembly, maturation, and replication. Previous study showed that avibirnavirus particles could be engulfed into the autophagosome and degradation of virus particles took apart. Selective autophagy is a highly specific and regulated degradation pathway for the clearance of damaged or unwanted cytosolic components and superfluous organelles as well as invading microbes. However, whether and how selective autophagy removes avibirnavirus capsids is largely unknown. Here, we have shown that selective autophagy specifically clears ubiquitinated avibirnavirus protein VP2 by p62 recognition and that p62 is an inhibitor of avibirnavirus replication, highlighting the role of p62 as a potential drug target for mediating the removal of ubiquitinated virus components from cells.

## Abstract (precis)

Avibirnavirus causes severe immunosuppression and mortality in young chickens. VP2, the capsid protein of avibirnavirus, is responsible for virus assembly, maturation, and replication. Previous study showed that avibirnavirus particles could be engulfed into the autophagosome and degradation of virus particles took apart. Selective autophagy is a highly specific and regulated degradation pathway for the clearance of damaged or unwanted cytosolic components and superfluous organelles as well as invading microbes. However, whether and how selective autophagy removes avibirnavirus capsids is largely unknown. Here, we have shown that selective autophagy specifically clears ubiquitinated avibirnavirus protein VP2 by p62 recognition and that p62 is an inhibitor of avibirnavirus replication, highlighting the role of p62 as a potential drug target for mediating the removal of ubiquitinated virus components from cells.

## INTRODUCTION

Autophagy is a highly conserved biological process that maintains cellular homeostasis by removing unwanted intracellular materials (1). Autophagy can be classed as selective or nonselective, where nonselective autophagy involves the bulk engulfment of cytoplasmic materials, and selective autophagy is responsible for specifically clearing certain cytoplasmic cargos, such as damaged or redundant organelles, invading pathogens, and protein aggregates (2–4). Accordingly, autophagy can limit or enhance viral replication (5, 6). In the case of antiviral autophagy, the selective targeting of viruses for degradation is a critical step. This step requires cargo receptors that mediate selective autophagy (7). The modular compositions of the interacting domains or motifs in cargo receptors enable them to link the selected materials to the appropriate sites of developing phagophores and autophagosomes. Several cargo receptors have been extensively characterized, such as p62, NBR1, and NDP52 (8–11). Most of these receptors recognize ubiquitinated substrates through their ubiquitin-associated domain (UBA) and then link to LC3 on nascent autophagosomes via the LC3-interacting region (LIR) (12–14).

The modification of cargos with ubiquitin is a well-known selective signal for autophagy. Ubiquitin, often labeled as simply Ub, is a highly conserved small protein with a molecular weight of 7 kDa that was first studied in the ubiquitin-proteasome system (UPS) (15). As a protein modifier, ubiquitin marks a target protein on lysine (Lys) residues in different forms under the action of three enzymes: E1, ubiquitin-activating enzyme; E2, ubiquitin-conjugating enzyme; and E3, ubiquitin protein ligase (16, 17). A single ubiquitin molecule (monoubiquitination) or many ubiquitin molecules (polyubiquitination) can be ligated to a Lys residue of the target protein (13, 18). Ubiquitin itself has several Lys residues that can be further ubiquitinated and then form polyubiquitin chains. Among the Lys residues available for further ubiquitination, K48 and K63 are the most extensively studied (19). Ubiquitination regulates both proteasomal and autophagy pathways. Generally, K63 chains seem to have a preferential affinity for autophagy cargo receptors, while proteins decorated with K48-, K27-, and K11-linked ubiquitin chains undergo proteasomal degradation (20, 21).

Avibirnavirus infectious bursal disease virus (IBDV) belongs to the *Birnaviridae* family (22, 23) and causes a highly contagious and immunosuppressive disease in young chickens. IBDV is a nonenveloped virus and contains two segments of double-stranded RNAs, including segment A and segment B (24, 25). The smaller segment B encodes VP1, the RNA-dependent RNA polymerase (26, 27). The larger segment A encodes VP5 and the polyprotein pVP2-VP4-VP3, which is autoproteolytically processed to generate precursor VP2 (pVP2), the viral protease VP4, and a major structural protein, VP3, through the proteolytic activity of VP4 (28, 29). Subsequently, pVP2 is further cleaved at its C-terminal end to the mature VP2 along with four short peptides (30, 31).

The IBDV capsid is a single-layer capsid composed of VP2 on the outer surface and VP3 on the inner surface (24, 32). VP2, as an avibirnavirus capsid, plays a key role in virus adsorption, virus invasion, and the induction of host immune response (33). Critical amino acid mutations in VP2 result in immune escape from vaccination against avibirnavirus (34–36).

The first link between autophagy and avibirnavirus was discovered by electron microscopy visualization in 1976, when the degradation of IBDV within autophagic vacuoles was observed (37). Since then, increasing evidence has demonstrated that avibirnavirus protein VP2 induces autophagy upon virus entry (33). However, whether autophagic induction degrades VP2 is still unclear.

Accordingly, in the present study we demonstrate that the avibirnavirus capsid protein VP2 is efficiently ubiquitinated at lysine residue 411 (K411). Thereafter, p62 recognizes the K411-ubiquitinated VP2 and delivers it to autophagosomes for degradation. Thus, our study highlights the role of p62 in mediating selective autophagy as a means to remove cytoplasmic pathogens.

## RESULTS

### The avibirnavirus capsid protein VP2 undergoes autophagic degradation.

**FIG 1:**
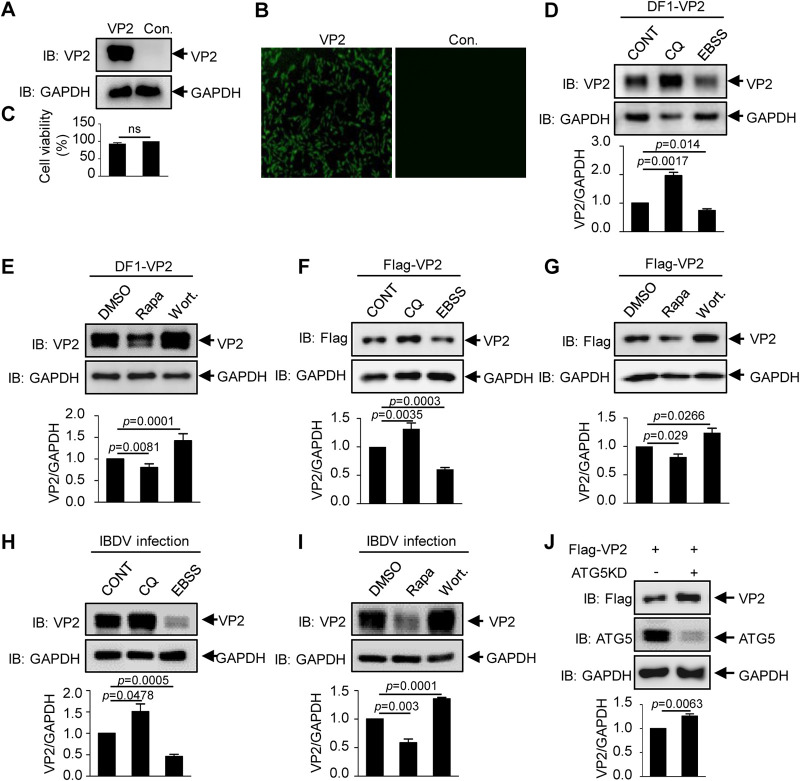
Avibirnavirus protein VP2 can be degraded via the autophagy pathway. (A) Western blot analysis of VP2 expression in VP2 stably expressed DF-1 cells. Con., control. (B) IFA results for VP2 stably expressed DF-1 cells. (C) Cell viability of VP2 stably expressed DF-1 cells. (D and E) VP2 stably expressed DF-1 cells were treated with 100 μM CQ, EBSS, 5 μM Rapa, and 20 nM Wort for 4 h. The cell lysates were subjected to immunoblot (IB) analysis normalized to GAPDH. (F and G) 293T cells transfected with Flag-VP2 for 24 h were treated with 100 μM CQ, EBSS, 5 μM Rapa, and 20 nM Wort for 4 h. The cell lysate was subjected to Western blotting normalized to GAPDH. (H and I) DF-1 cells were pretreated with 100 μM CQ, EBSS, 5 μM Rapa, and 20 nM Wort for 4 h, and then the cells were infected with IBDV at an MOI of 10 and cultured for 4 h. The cell lysates were subjected to immunoblot analysis normalized to GAPDH. (J) Flag-VP2 was transfected into WT or *ATG5* KD 293T cells. At 24 h posttransfection, the cell lysate was subjected to immunoblot analysis with the indicated antibodies. Data are presented as means ± SD from at least three independent experiments. ns, *P* > 0.05; *, *P* < 0.05; **, *P* < 0.01; ***, *P* < 0.001.

A previous study demonstrated that the degradation of IBDV particles in autophagic vacuoles and the binding of viral protein VP2 to HSP90AA1 activate autophagy by the AKT-MTOR pathway in the early stage of infection (33). Accordingly, we investigated whether VP2, the major capsid protein of avibirnavirus, is subject to autophagic degradation via autophagic induction. The *VP2* gene was inserted into lentivirus vector PCDH-CMV-MCS-EF1 to construct a DF-1 cell line that stably expresses VP2. Immunoblot assay and immunofluorescence assay (IFA) using anti-VP2 monoclonal antibody revealed that viral protein VP2 is successfully expressed in the VP2 stably expressed DF-1 cells (Fig. 1A and B). Cell viability assays showed that the activity of the VP2 stably expressed cells is not influenced significantly compared to that of wild-type (WT) DF-1 cells (Fig. 1C). Subsequently, we tested the levels of VP2 in VP2 stably expressed DF-1 cells and 293T cells transiently transfected with Flag-VP2 in the presence or absence of an autophagy inducer or inhibitor. Western blot assays (WB) showed that the autophagy inhibitors wortmannin (Wort) and chloroquine (CQ) significantly increase the level of VP2, while the autophagy activators rapamycin (Rapa) and Earle’s balanced salt solution (EBSS) markedly decrease the level of VP2 (Fig. 1D to G). Similarly, the level of VP2 was also measured in avibirnavirus-infected cells treated with autophagy inducer or inhibitor. The results showed that CQ or Wort treatment inhibited the degradation of VP2, while EBSS or Rapa treatment promoted the degradation of VP2 (Fig. 1H and I), confirming that autophagy regulated the degradation of VP2 produced by avibirnavirus. In addition, we further investigated the level of VP2 in 293T cells with ATG5 knockdown (KD), which is required for autophagy (38). The level of VP2 was shown to increase greatly in *ATG5* KD 293T cells compared with that in WT 293T cells (Fig. 1J). These data indicate that autophagy plays a critical role in the degradation of the IBDV capsid protein VP2.

### Autophagic induction enhances VP2 interaction with the cargo receptor p62.

**FIG 2:**
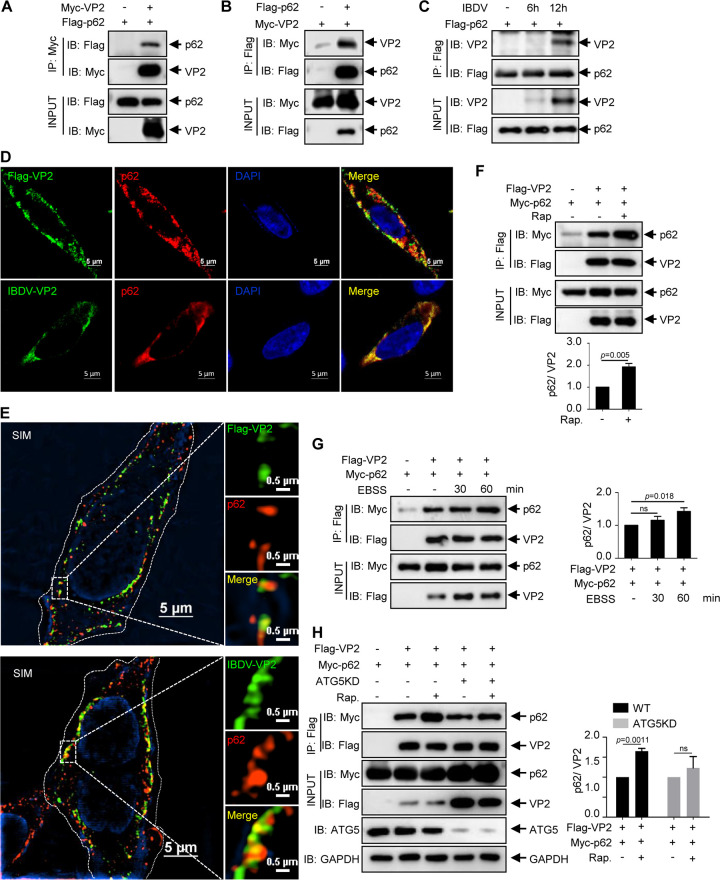
VP2 interacts with cargo receptor p62. (A) 293T cells were cotransfected with Flag-p62 and Myc-VP2 for 36 h. The cell lysate then was subjected to coimmunoprecipitation of anti-Myc MAb and immunoblot analysis with anti-Myc and anti-Flag antibodies. (B) 293T cells were cotransfected with Myc-VP2 and Flag-p62 and subjected to co-IP of anti-Flag antibody and immunoblot analysis. (C) DF-1 cells transfected with Flag-p62 for 24 h were infected with IBDV at an MOI of 0.1 for 0, 6, and 12 h. Co-IP and Western blot assays were performed using anti-Flag and anti-VP2 antibodies. (D) Flag-VP2 was transfected into DF-1 cells for 24 h, and fresh DF-1 cells were infected with IBDV at an MOI of 0.1 for 24 h. The cells were incubated with mouse anti-Flag or mouse anti-VP2 antibodies and rabbit anti-p62 antibody, followed by staining with FITC goat anti-mouse and Alexa Fluor 546 anti-rabbit as the secondary antibodies. The nuclei were stained with DAPI. The fluorescence signals were observed and imaged using confocal microscopy. Scale bar, 5 μm. (E) The fluorescence signals of cell samples from panel D were observed and imaged using 3D-SIM microscopy, and raw images of SIM were reconstructed using NIS Elements. Scale bar, 5 or 0.5 μm. (F and G) 293T cells were transfected with Myc-p62 and Flag-VP2 for 36 h. The cells were then treated with or without 0.5 μM Rapa for 6 h (F) and with EBSS for 0, 30, and 60 min (G). Co-IP and immunoblot analyses were performed using anti-Flag, anti-Myc, and anti-VP2 antibodies. (H) WT or *ATG5* KD 293T cells were transfected with Myc-p62 and Flag-VP2 for 36 h. The cells were left untreated or were treated with 0.5 μM Rapa for 6 h for co-IP and immunoblot analyses. Results are presented as means ± SD from at least three independent experiments. ns, *P* > 0.05; *, *P* < 0.05; **, *P* < 0.01.

Increasing evidence supports the idea that cargo receptors, especially p62, deliver viral proteins to autophagosomes for selective degradation during infection (39–41). Accordingly, to investigate the relationship between cargo receptor p62 and viral protein VP2, 293T cells were cotransfected with vectors expressing Myc-VP2 and Flag-p62 for 36 h (or transfected with Flag-p62 or Myc-VP2 alone as negative controls). Coimmunoprecipitation (co-IP) was then performed with anti-Myc or anti-Flag antibody, respectively. Subsequent co-IP assays showed that Flag-p62 interacts with Myc-VP2 (Fig. 2A and B).

We also investigated viral protein VP2 interaction with p62 during IBDV infection. DF-1 cells transfected with Flag-p62 for 24 h were infected with avibirnavirus for 0, 6, or 12 h. As shown in Fig. 2C, viral protein VP2 in avibirnavirus-infected cells also interacts with p62. Moreover, confocal and structured illumination microscopy (SIM) indicated that VP2 colocalized with endogenous p62 in VP2 transfected cells or avibirnavirus-infected cells (Fig. 2D and E). Since the autophagy inducer promotes the degradation of VP2, we investigated whether the autophagy inducer affects the interaction of viral protein VP2 and p62. Anti-Flag IP and immunoblot analysis showed that Rapa and EBSS treatment increase the affinity of VP2 for p62 (Fig. 2F and G). To further verify the effect of autophagy on the VP2 interaction with p62, Flag-VP2 and Myc-p62 were cotransfected into WT or *ATG5* KD 293T cells with or without Rapa treatment. Anti-Flag IP showed that the knockdown of ATG5 blunted the effect of Rapa on the affinity of VP2 to p62, suggesting that autophagy itself indeed regulated the VP2 interaction with p62 (Fig. 2H). Thus, the data described above collectively demonstrate that autophagy induction promotes the binding of p62 to VP2.

### *p62* is involved in the selective autophagic degradation of VP2.

**FIG 3:**
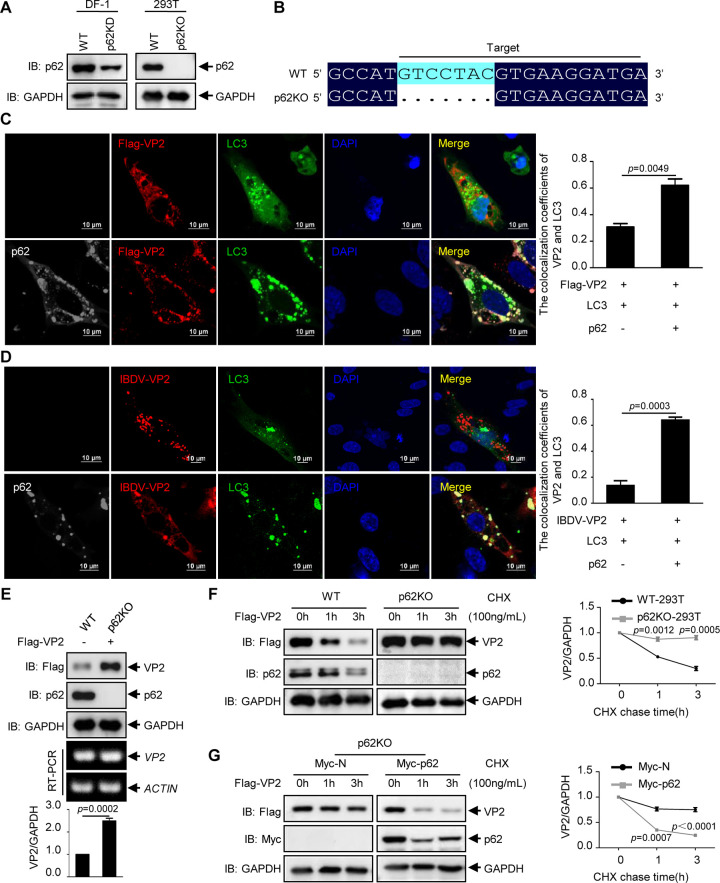
p62 is involved in the autophagic degradation of VP2. (A) Western blot analysis of p62 expression in *p62* KD DF-1 cells and monoclonal *p62* KO 293T cells. (B) Gene sequence comparison of WT and *p62* KO 293T cells. (C) Flag-VP2 and EGFP-LC3 were cotransfected with or without Myc-p62 into *p62* KD DF-1 cells for 24 h. The cells were incubated with mouse anti-Flag antibody and rabbit anti-Myc antibody, followed by the indicated secondary antibodies. The stained cells were observed using confocal microscopy. Quantitative analysis of the colocalization coefficient between VP2 and GFP-LC3 was performed with NIS Elements (Nikon, Japan). Scale bar, 10 μm. (D) EGFP-LC3 was transfected with or without Myc-p62 into *p62* KD DF-1 cells for 24 h, followed by IBDV infection at an MOI of 0.1 for 24 h. The cells were incubated with mouse anti-VP2 antibody and rabbit anti-p62 antibody, followed by the indicated secondary antibodies. The fluorescence signals were observed using confocal microscopy. Scale bar, 10 μm. (E) Flag-VP2 was transfected into WT 293T and *p62* KO 293T cells for 24 h. Immunoblotting with the indicated antibodies and RT-PCR was performed. (F) Flag-VP2 was transfected into WT or *p62* KO 293T cells. At 24 h posttransfection, the cells were treated with CHX (100 ng/ml) for 0, 1, or 3 h. The cell lysate was subjected to immunoblot analysis with the indicated antibodies. (G) Flag-VP2 was cotransfected with an empty vector or Myc-p62 into *p62* KO 293T cells. At 24 h posttransfection, the cells were treated with CHX (100 ng/ml) for 0, 1, or 3 h. The cell lysate was subjected to Western blotting with the indicated antibodies. Results are presented as the means ± SD from at least three independent experiments. **, *P* < 0.01; ***, *P* < 0.001; ****, *P* < 0.0001.

We next investigated whether p62 functions as a cargo receptor to mediate the autophagic degradation of VP2. Therefore, *p62* knockdown DF-1 (*p62* KD) and *p62* knockout (*p62* KO) 293T cell lines were generated using Cas9 enzyme (Fig. 3A and B), and the distribution of VP2 in the *p62* KD and KO cells was determined. As shown in Fig. 3C and D, viral protein VP2 did not overlap GFP-LC3 in *p62* KD cell lines, while the expression of p62 promoted colocalization between VP2 and GFP-LC3.

Since p62 promoted the localization of VP2 at autophagosomes, we next investigated whether p62 affects the level of VP2. Flag-VP2 was transfected into WT and *p62* KO 293T cell lines for 24 h. As shown in Fig. 3E, the level of VP2 mRNA was not significantly different between WT and *p62* KO 293T cells, but the level of VP2 protein was much higher in *p62* KO cells.

In addition, we conducted a cycloheximide (CHX) chase assay to determine the degradation of VP2. WT and *p62* KO 293T cells were transfected with Flag-VP2 for 24 h and treated with 100 ng/ml CHX for 0, 1, or 3 h. As shown in Fig. 3F, the Western blot assay shows that the degradation of VP2 is significantly inhibited in *p62* KO cells. Furthermore, the *p62* KO cell line was cotransfected with Flag-VP2 and Myc-p62 for 24 h, and a CHX chase assay was then performed. The results show that the expression of p62 rescues the degradation of VP2 (Fig. 3G). Collectively, these data demonstrate that p62 promotes the autophagic degradation of VP2.

### Both the UBA and LIR domains are required for p62-mediated degradation of VP2.

**FIG 4:**
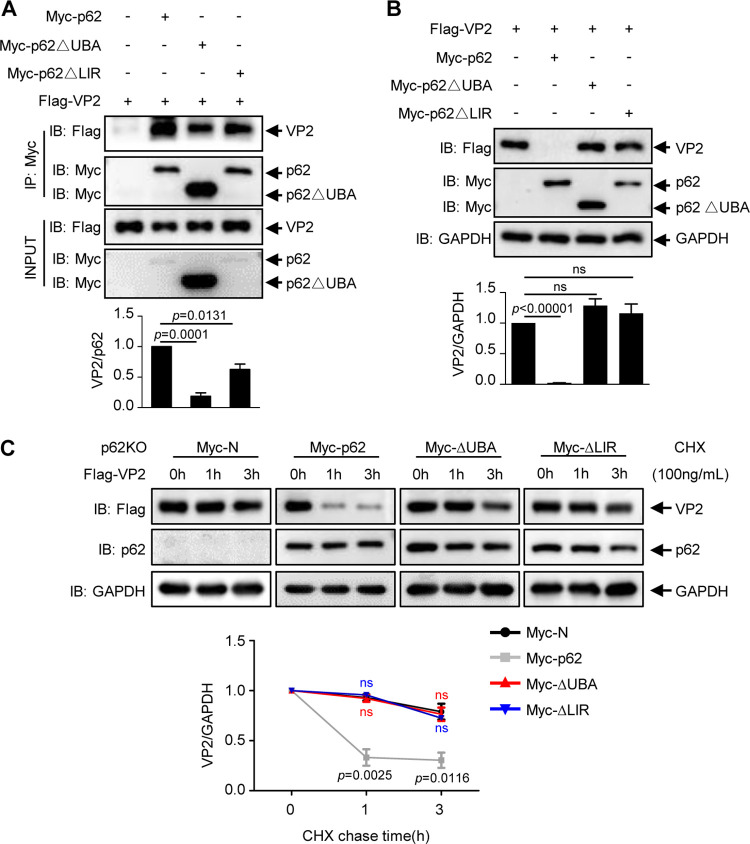
UBA and LIR domains of p62 are required for degrading viral protein VP2. (A) Flag-VP2 was transfected alone or cotransfected with Myc-p62, Myc-p62-△UBA, or Myc-p62-△LIR into 293T cells for 36 h. Cell lysates were subjected to coimmunoprecipitation using an anti-Myc MAb and immunoblot analysis using the indicated antibodies. (B) The cell lysates from panel A were subjected to immunoblot analysis with the indicated antibodies. (C) Flag-VP2 was cotransfected with Myc-N, Myc-p62, Myc-p62-△UBA, or Myc-p62-△LIR into *p62* KO 293T cells. At 24 h posttransfection, the cells were treated with CHX (100 ng/ml) for 0, 1, or 3 h. The cell lysate was subjected to immunoblot analysis with the indicated antibodies. Results are presented as the means ± SD from at least three independent experiments. ns, *P* > 0.05; *, *P* < 0.05; **, *P* < 0.01; ***, *P* < 0.001; ****, *P* < 0.0001.

p62 utilizes the UBA domain to bind to ubiquitinated substrates and the LIR domain to interact with LC3, subsequently linking substrates to LC3-decorated autophagosomes (8, 42). Therefore, we constructed the mutants Myc-p62△UBA and Myc-p62△LIR to investigate whether p62 interacts with VP2 through the UBA domain and links VP2 to LC3 through the LIR domain. Flag-VP2 was cotransfected with Myc-p62 or the mutants into 293T cells. Co-IP assays showed that the deletion of the UBA domain (Myc-p62△UBA) significantly decreases the interaction with VP2 (Fig. 4A), suggesting that the UBA domain of p62 is necessary for such interaction.

We also investigated whether the UBA domain or LIR domain is required for the p62-mediated degradation of VP2. We found that neither the UBA-deficient mutant nor the LIR-deleted mutant of p62 promotes the degradation of VP2 (Fig. 4B), suggesting that both UBA and LIR domains are required for p62-mediated degradation of VP2. Subsequently, we further conducted a CHX chase assay to test whether the UBA domain or LIR domain influences the degradation of VP2 in *p62* KO 293T cells. As shown in Fig. 4C, compared to p62, the UBA-deleted mutant and the LIR-deleted mutant could not promote the degradation of VP2, confirming that both UBA and LIR of p62 were necessary for the degradation of VP2.

Thus, the results described above indicate that binding by both the UBA and LIR domains of p62 to VP2 and LC3, respectively, is necessary for autophagic degradation of VP2, confirming that p62 acts as a cargo receptor linking VP2 to autophagosomes.

### The ubiquitination of VP2 at lysine 411 is required for p62-mediated autophagic degradation.

Since the UBA domain is necessary for the interaction of p62 with VP2, we hypothesized that VP2 binding to p62 is involved in ubiquitination. Accordingly, we first investigated VP2 ubiquitination.

**FIG 5:**
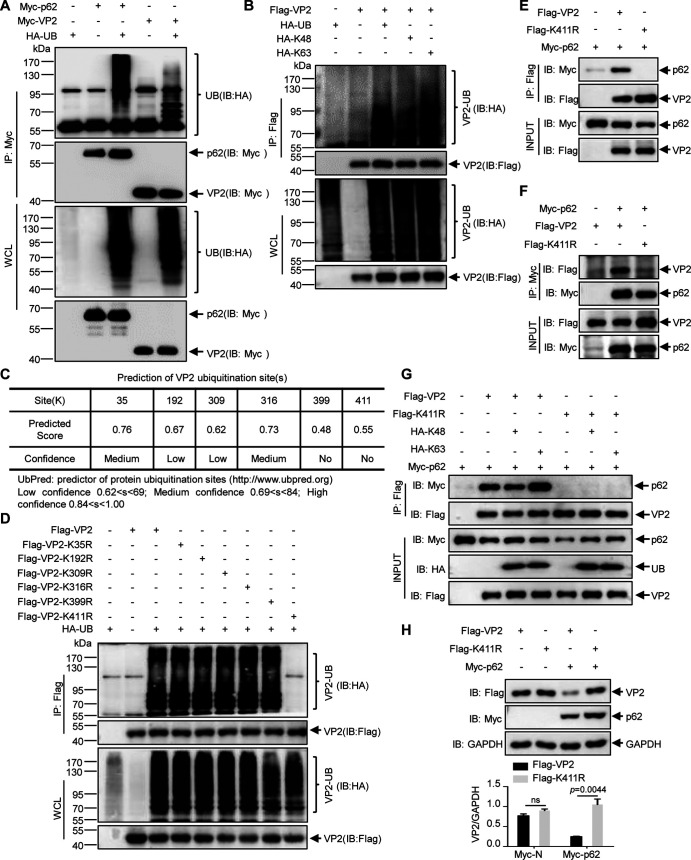
K63-linked ubiquitination of VP2 is required for p62-mediated degradation. (A) Myc-VP2 was cotransfected with HA-Ub into 293T cells for 36 h, and Myc-p62 was cotransfected with HA-Ub as a positive control. Cell lysates were subjected to ubiquitination analysis and Western blotting. WCL, whole-cell lysate. (B) Flag-VP2 was cotransfected with HA-Ub, HA-K48, or HA-K63 into 293T cells for 36 h. Cell lysates were subjected to ubiquitination analysis and Western blotting. (C) Potential ubiquitination sites in VP2 were predicted in UbPred (http://www.ubpred.org). (D) Flag-VP2 or the K-to-R mutants of VP2 were cotransfected with HA-Ub into 293T cells for 36 h. Ubiquitination assays and Western blotting were then performed. (E) 293T cells were cotransfected with Flag-VP2 or Flag-K411R and Myc-p62. Cell lysates were subjected to coimmunoprecipitation assay with anti-Flag MAb and immunoblot analysis with the indicated antibodies. (F) 293T cells were transfected with Flag-VP2 or Flag-K411R and Myc-p62. The indicated antibodies were used for coimmunoprecipitation of anti-Myc and immunoblot assays. (G) Flag-VP2 or Flag-K411R and Myc-p62 were cotransfected with or without HA-K48 or HA-K63 into 293T cells for 36 h. Cell lysates were subjected to coimmunoprecipitation assay with anti-Flag MAb and immunoblot analysis with the indicated antibodies. (H) Flag-VP2 or Flag-K411R was transfected into 293T cells for 24 h in the absence or presence of Myc-p62 expression. The indicated antibodies were used for immunoblot assays. Results are presented as the means ± SD from at least three independent experiments. ns, *P* > 0.05; **, *P* < 0.01.

Myc-VP2 and hemagglutinin (HA)-Ub were transfected separately or together into 293T cells for 36 h, and then ubiquitination assays were conducted. Myc-62 and HA-Ub were cotransfected into cells as a positive control (43). Co-IP assays revealed that the viral protein VP2 and p62 were ubiquitinated (Fig. 5A). Further study showed that VP2 was ubiquitinated predominantly via K63-linked rather than K48-linked ubiquitination (Fig. 5B). To further identify the ubiquitination site of VP2, we predicted the sites online. As shown in Fig. 5C, there are six potential ubiquitination sites in VP2. These were each separately mutated from lysine (K) to arginine (R), and the mutants then were subjected to *in vivo* ubiquitination assays. As shown in Fig. 5D, only mutation of K411R abrogates the ubiquitination. Moreover, p62 no longer interacted with the K411R mutant of VP2 (Fig. 5E and F). To investigate whether the ubiquitin chain affects the p62 interaction with viral protein VP2, Flag-VP2 or Flag-K411R and Myc-p62 were cotransfected together with or without HA-K48 or HA-K63 into 293T cells. Coimmunoprecipitation assay with anti-Flag MAb showed the K63 ubiquitin chain increased the affinity of wild-type VP2 rather than the K411R mutant to p62 (Fig. 5G). These data suggest that K411 with K63 ubiquitination is required for the interaction of VP2 with p62.

We next investigated whether the K411 ubiquitination of VP2 is required for p62-mediated degradation. 293T cells were cotransfected with Flag-VP2, Flag-VP2-K411R and Myc-N, or Myc-p62. Western blot analysis revealed that the K411R mutant of VP2 is resistant to degradation, unlike WT VP2, in the case of p62 overexpression (Fig. 5H). Thus, the data suggest that the p62-mediated degradation of VP2 depends on its ubiquitination at K411.

### p62 inhibits avibirnavirus replication.

**FIG 6:**
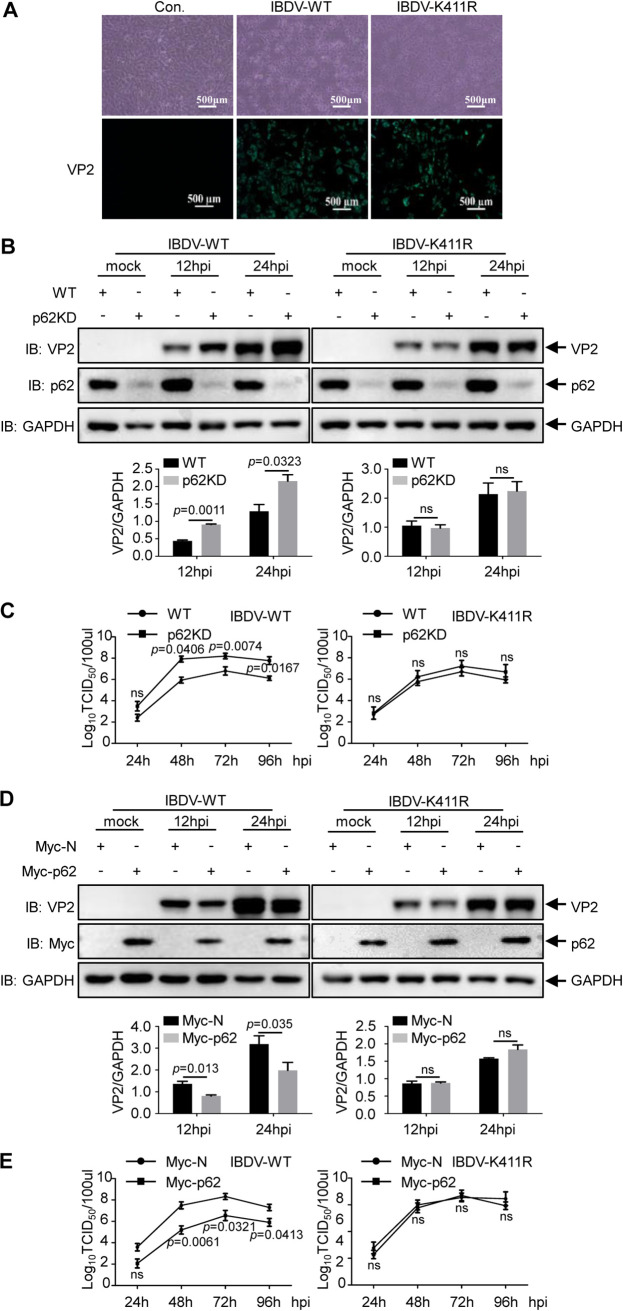
p62 blocks avibirnavirus replication. (A) Rescue of mutant IBDV-K411R using the IBDV strain CT rescue system. DF-1 cells were infected with IBDV-WT or IBDV-K411R. CPE was observed by a phase-contrast microscope, and the infected cells were incubated with anti-VP2 MAb and FITC anti-mouse IgG as the primary antibody and the secondary antibody, respectively. The fluorescence signals of VP2 were observed with an inverted fluorescence microscope (Nikon, Japan). Scale bar, 500 μm. (B) WT or *p62* KD DF-1 cells were infected with IBDV-WT or IBDV-K411R at an MOI of 0.1 for 0, 12, and 24 h and subjected to immunoblot analysis with the indicated antibodies. (C) One-step growth curve of IBDV. WT or *p62*KD DF-1 cells were infected with IBDV-WT or IBDV-K411R at an MOI of 0.1 at the indicated times, and the TCID_50_ was detected as described in Materials and Methods. (D) DF-1 cells transfected with Myc-N or Myc-p62 were infected with IBDV-WT or IBDV-K411R at an MOI of 0.1 for 0, 12, and 24 h. The level of VP2 protein was determined by immunoblotting. (E) Myc-N or Myc-p62 was transfected into DF-1 cells, followed by IBDV-WT or IBDV-K411R infection at an MOI of 0.1 for the indicated times. TCID_50_ assay was performed to draw the growth curve of IBDV-WT and IBDV-K411R. Results are presented as the means ± SD from at least three independent experiments. ns, *P* > 0.05; *, *P* < 0.05; **, *P* < 0.01.

Given that p62 mediates the degradation of avibirnavirus protein VP2, whether p62 inhibits avibirnavirus replication is unknown. To further investigate the effect of p62 on avibirnavirus replication, the rescue of ubiquitination-deficient VP2 IBDV (IBDV-K411R) was performed by the T7 expression system. Rescued virus was verified by observing cytopathic effects (CPEs) and the fluorescence signals of capsid protein VP2 under the microscope (Fig. 6A). We then determined the level of VP2 and the avibirnavirus titer in p62-overexpressing and p62 KD cells infected with IBDV-WT or IBDV-K411R. As shown in Fig. 6B, the VP2 level significantly increased in IBDV-infected *p62* KD DF-1 cells, while the level of VP2 had no significant change in IBDV-K411R-infected *p62* KD DF-1 cells. Furthermore, virus replication assays showed that, compared to wild-type IBDV, the replication ability of IBDV-K411R no longer remarkably increased in *p62* KD DF-1 (Fig. 6C). Consistent with this result, we found that the overexpression of p62 in DF-1 cells decreased the wild-type IBDV replication rather than IBDV-K411R replication (Fig. 6D and E). Collectively, these results demonstrate that p62 represses the replication of wild-type but not K411R mutant avibirnavirus, confirming that p62 is an avibirnavirus inhibitor via degrading capsid protein VP2.

## DISCUSSION

Selective autophagy has an important function in the immune system, where it plays an antiviral role by removing viral components. Some evidence indicates that autophagy restricts viruses by removing cytoplasmic viral components, genomes, or viral particles (44–46). As selected examples, Smad ubiquitin regulatory factor 1 (SMURF1), an E3-ubiquitin ligase, is required for the p62-dependent autophagic degradation of Sindbis virus (SINV) capsid protein (47); Fanconi anemia group C protein has been reported to interact with the SINV capsid protein and to promote its autophagic degradation (41, 48); Homo sapiens Shisa family member 5 restricts hepatitis C virus replication by targeting the viral nonstructural protein 5A (NS5A) for autophagic degradation (46); and histone deacetylase 6 inhibits human immunodeficiency virus 1 replication by forming a complex with apolipoprotein B mRNA-editing enzyme and catalytic polypeptide-like 3G and promoting autophagy-dependent Vif degradation (49). In addition, galectin 8 suppresses viral infection by autophagic degradation of the viral RNA genome (45). Avibirnavirus VP2 is also reported to undergo lysosomal degradation (50).

In the present study, we revealed that p62-mediated selective autophagy degrades the avibirnavirus capsid protein VP2 in a ubiquitin-dependent manner. Thus, our findings provide the first evidence that autophagy recognizes and degrades virus capsid proteins in a ubiquitin-dependent manner.

p62 is a well-known autophagy cargo receptor and contains the UBA domain and an LC3 interaction region, both of which recognize ubiquitinated substrates and deliver them to autophagosomes for final degradation in the lysosome (8, 51). Recently, the regulation of the selective autophagic degradation of the SIN virus was reported to involve p62 binding to its capsid. The same study demonstrated that the knockdown of p62 blocks GFP-LC3 colocalization with the SIN capsid and blunts the ability of autophagy to remove the SIN capsid from infected cells (47). However, whether recognition of the SIN capsid by p62 involves ubiquitination is unclear. However, in the current study, we have demonstrated that p62 binds to avibirnavirus capsid VP2 and that this binding is dependent on the UBA domain of p62 and the ubiquitination of VP2. Either the UBA or LIR domain, as well as the ubiquitination of VP2, is required for the p62-mediated degradation of VP2, suggesting that p62 acts as a bridge connecting VP2 to the autophagosome for degradation.

Autophagy is critical for antibody production (52, 53). In fact, avibirnavirus VP2 induces the production of neutralizing antibodies (54, 55). Currently, the IBDV subunit vaccine and recombinant vaccine against avibirnavirus are designed based on VP2 (56–58). In addition, the autophagic regulation of antigen processing and presentation is critical for CD4^+^ and CD8^+^ T cell activation (59–66). Conjugating the human immunodeficiency virus 1 antigen to p62 enhances T-cell-mediated immunity (5). Viral protein VP2, the capsid of avibirnavirus, plays a key role in viral adsorption and viral core assembly and is one of its main immunogens. Our earlier study showed that autophagy can be induced by VP2 at the stage of avibirnavirus entry (33) and that autophagy is inhibited by viral VP3 at a later stage of infection (29). In our present study, we have found that lysine residue 411 of VP2 is modified in avibirnavirus-infected cells in a K63-linked ubiquitination manner and degraded by binding to p62. Thus, we hypothesize that p62-mediated autophagic degradation of VP2 participates in the presentation of VP2 peptide to initiate the adaptive immune response against avibirnavirus. Thus, pharmacological control of p62 expression represents a potential strategy for the treatment of avibirnavirus-induced diseases.

In summary, our study has demonstrated that viral protein VP2 is a K63-linked ubiquitination protein that binds to cargo receptor p62. Our data provide strong evidence for p62-mediated selective autophagic degradation of VP2 against IBDV replication, highlighting the antiviral role of p62-mediated autophagy.

## MATERIALS AND METHODS

### Cells and virus.

Human embryonic kidney (HEK) 293T cells (referred to as 293T cells) were cultured in Dulbecco’s modified Eagle’s medium (DMEM; Gibco, Carlsbad, CA, USA) supplemented with 10% fetal bovine serum (FBS) (F2442-500ML; Sigma, St. Louis, MO, USA). Chicken embryo fibroblasts (DF-1) were grown in DMEM containing 10% FBS (CCS30010.02; MRC, QLD, Australia). The T7 RNA polymerase stably expressing cell line, BSRT7 (kindly gifted by Jingjing Cao, Shandong University, China), was cultured in DMEM containing 10% FBS (F2442-500ML; Sigma, St. Louis, MO, USA) and 500 μg/ml G418 (A100859; Sangon Biotech, Shanghai, China). Avibirnavirus IBDV strain NB is maintained in our laboratory (27).

### Antibodies and reagents.

VP2 mouse monoclonal antibody (MAb) was produced in our laboratory (33). Mouse MAb against Flag (F1804) and HA (H9658) were purchased from Sigma-Aldrich (St. Louis, MO, USA). Mouse anti-Myc MAb was purchased from Millipore (MABE282). Rabbit polyclonal antibodies (pAb) against Myc (R1208-1) and glyceraldehyde-3-phosphate dehydrogenase (GAPDH) (EM1101) were obtained from Huaan Biological Technology (Hangzhou, China). Rabbit anti-SQSTM1 pAb was purchased from Epitomics (3340-1; Burlingame, CA, USA). Rabbit anti-ATG5 pAb was purchased from ABclonal Technology (A0203; Wuhan, China). Horseradish peroxidase (HRP)-labeled anti-mouse or anti-rabbit IgG (074-1806 and 074-1506) for Western blotting and fluorescein isothiocyanate (FITC)-labeled goat anti-mouse IgG (172-1806) were purchased from KPL (Millford, MA, USA). Alexa Fluor 546-conjugated anti-mouse (A10036), anti-rabbit (A21085) IgG, and Alexa Fluor 647-labeled donkey anti-mouse IgG (A21236) were purchased from Invitrogen (Carlsbad, CA, USA). Cell lysis buffer NP-40 (50 mM Tris [pH 7.4], 150 mM NaCl, 1% NP-40) was purchased from Beyotime (P0013F; Shanghai, China). Protein A/G plus-agarose, for coimmunoprecipitation assays, was obtained from Santa Cruz Biotechnology (D2117; Santa Cruz, CA, USA). Chloroquine (C6628), rapamycin (R8781), wortmannin (681675), and *N*-ethylmaleimide (E3876) were purchased from Sigma-Aldrich. Puromycin (ant-pr) was purchased from Invivogen.

### Plasmid construction and transfection.

The IBDV *VP2* gene from segment A was cloned into the pCMV-Flag-N vector (635688; Clontech, Mountain View, CA) and the pCMV-Myc-N vector (635689; Clontech, Mountain View, CA). All mutants of pCMV-Flag-VP2 and rescue plasmid T7-A were constructed by site-directed mutagenesis technology. Expression plasmid pEGFP-LC3B was constructed in our laboratory (33). The human *p62* gene was amplified by PCR from total cellular RNA with gene-specific primers (5′-GCGAATTCGCATGGCGTCGCTCACC-3′ and 5′-GCTCTAGAGCTCACAACGGCGGGGG-3′) and subcloned into the pCMV-Flag-N vector and the pCMV-Myc-N vector. The mutants of Myc-p62, Myc-p62△UBA, and Myc-p62-△LIR were constructed with special primers. The primers 5′-TTGTACCCACATCTCCCCCCGCCGTTGTGA-3′ and 5-TCACAACGGCGGGGGGAGATGTGGGTACAA-3′ were used for Myc-p62△UBA. The primers 5′-GAGGAGATGATGACTCTTCAAAAGAAGT-3′ and 5′-ACTTCTTTTGAAGAGTCATCATCTCCTC-3′ were used for Myc-p62△LIR. HA-Ub, HA-K48, and HA-K63 expression plasmids were kindly gifted by Hongbin Shu (College of Life Sciences, Wuhan University). All expression plasmids were transfected into 293T cells and DF-1 cells using Lipofectamine 3000 reagent (L3000015; Life Technologies, Carlsbad, CA, USA) according to the manufacturer’s instructions.

### Coimmunoprecipitation, ubiquitination, and Western blot assays.

For coimmunoprecipitation assays, 293T cells were prepared in six-well plates and cultured for 8 to 12 h until 70% to 90% confluence before transfection by Lipofectamine 3000 reagent. After 36 to 48 h of transfection, the resultant cells were harvested and lysed at 4°C for 2 h by NP-40 lysis buffer supplemented with 1 mM phenylmethylsulfonyl fluoride (PMSF). After centrifugation at 12,000 × *g* for 10 min at 4°C, the supernatant was collected and treated with 20 μl protein A/G plus-agarose at 4°C for 30 min to remove impurities. After centrifugation at 1,000 × *g* for 5 min at 4°C, the supernatant was incubated with anti-Flag or anti-Myc mouse MAb at 4°C for 2 h. A volume of 80 μl protein A/G plus-agarose then was added to these immune complexes, which were incubated at 4°C for 2 h. Finally, the beads were washed with NP-40 five times at 4°C and boiled in 4× protein loading buffer (with sodium dodecyl sulfate, dl-dithiothreitol, and bromophenol blue) for 10 min. The samples were then subjected to Western blot assay.

For ubiquitination assays, Flag-VP2 and HA-Ub were cotransfected into 293T cells for 36 to 48 h. The cells were then harvested and lysed using NP-40 containing PMSF and 5 mM *N*-ethylmaleimide at 4°C for 2 h. Immunoprecipitation and Western blot assays were then performed.

For Western blotting, the cell samples were subjected to SDS-PAGE after centrifugation at 12,000 × *g* for 10 min at room temperature and then transferred to a nitrocellulose membrane. After blocking with 5% skimmed milk, the membrane was incubated with the indicated primary antibodies at room temperature for 2 to 4 h or at 4°C overnight. The membrane then was washed with phosphate-buffered saline (PBS) containing 0.1% Tween 20 (PBST) five times and incubated with HRP-labeled secondary antibody at room temperature for 1 to 2 h. The immunoreactive protein bands were detected and imaged using enhanced chemiluminescence and a chemiluminescence imaging system, respectively.

### Virus infection.

DF-1 cells were cultured on 6- or 12-well plates and infected with IBDV diluted in DMEM at a multiplicity of infection (MOI) of 0.01 or 10. After 1 h of absorption at 37°C, the medium was removed. The infected cells then were washed with PBS three times and cultured in fresh DMEM containing 2% FBS at 37°C until the specified time.

### Cell viability assay.

WT DF-1 and VP2 stably expressed DF-1 cells were cultured on 96-well plates with 5,000 cells per well for 24 h. Blank wells (medium without cells) were added with the same amount of culture medium, and then 10 μl/well (96-well plate) CCK8 solution was added to each well. The treated cells were protected from light and incubated for 2 h at 37°C. The absorbance increase at 450 nm was measured.

### Indirect immunofluorescence assay and confocal microscopy.

To assess the colocalization of VP2, p62, and LC3, DF-1 cells were seeded in confocal dishes and cotransfected with Flag-VP2 and Myc-p62 with or without EGFP-LC3. After 24 to 36 h of transfection, cells were fixed using 4% paraformaldehyde for 10 min and permeabilized with 0.2% Triton X-100 for 5 min at room temperature. The fixed cells were incubated with primary anti-Flag and anti-Myc antibodies diluted in 5% skimmed milk for 4 h at 37°C or at 4°C overnight. The cells then were washed with PBS four times and incubated with the corresponding secondary antibodies for 2 h at 37°C. The cells next were stained with 4ʹ,6-diamidino-2-phenylindole (DAPI) for 10 min at room temperature. Finally, the cells were scanned using a Nikon A1R/A1 laser scanning confocal microscope (Nikon, Tokyo, Japan).

### Reverse transcription-PCR (RT-PCR).

293T cells were transfected with Flag-VP2 for 24 h. Total cellular RNA was extracted by the TRIzol reagent (R401-01; Vazyme Biotech Co., Ltd.) by following the manufacturer’s instructions. Reverse transcription of extracted RNA was conducted using a reverse transcription kit (R211-01; Vazyme Biotech Co., Ltd.) according to the manufacturer’s instructions. The cDNA was amplified by the specific primers. The primers 5′-GTCAGCCGCATCTTCTTTTG-3′ and 5′-GCGCCCAATACGACCAAATC-3′ were used for *ACTIN*. The primers 5′-AATGGTAGCCACATG-3′ and 5′-GCCTGACCACCACTT-3′ were used for *VP2*.

### CHX chase assay.

The indicated expression plasmids were transfected into 293T cells. After 24 h of transfection, the cells were treated with 100 ng/ml CHX dissolved in dimethyl sulfoxide (DMSO). The cells then were harvested at various time points. Finally, the samples were subjected to Western blot analysis. ImageJ software and GraphPad software were used to analyze the protein levels.

### Generation of KD and KO cells.

The *p62* gene target sequence, 5′-TAACTTACCATAGACATCTG-3′, and the *ATG5* gene target sequence, 5′-AACTTGTTTCACGCTATATC-3′, were inserted into the guide RNA expression plasmid PX459 (62988; Addgene, Watertown, MA, USA), a vector expressing Cas9 enzyme. The constructed plasmid was transfected into HEK293T cells for 48 h, and then the positive cells were selected under 4 μg/ml puromycin conditions for another 72 h. The cells were then diluted to 50 cells/100 ml and inoculated into 96-well plates for colony formation. Each colony was separately transferred into 24-well plates. KO and KD of p62 or KD of ATG5 was confirmed by Western blotting.

### Construction of DF-1 cell lines with stable expression of VP2.

The *VP2* gene was inserted into overexpression lentivirus vector PCDH-CMV-MCS-EF1 (CD510B-1; System Biosciences, Palo Alto, CA, USA). The constructed plasmid was transiently transfected into DF-1 cells, and Western blotting was used to detect successful expression. The plasmid then was cotransfected with the ViraPower lentiviral packaging mix (K497500; Invitrogen) into 293T cells to produce a lentiviral stock according to the manufacturer’s instructions. An empty vector was used for the control. After 72 h of transfection, viral particles were harvested from the medium by ultracentrifugation. After lentiviral preparation, DF-1 cells were seeded into a six-well plate and grown overnight to 80% confluence. The next day, cells were separately infected with 2 ml PCDH-CMV-VP2-EF1 and PCDH-CMV-MCS-EF1 virus for 6 h. After transduction, cells were cultured for another 24 h with complete medium and then screened for positive cells using 2 μg/ml puromycin. The expression of VP2 in the cell lines was detected by IFA and Western blotting. Cell viability was determined using a CCK8 kit (C0038; Beyotime Biotechnology, Shanghai, China).

### Virus rescue.

The mutant strain VP2-K411R was produced by using the IBDV strain CT rescue system. Rescue plasmid T7-B was transfected alone or cotransfected with rescue plasmid T7-A or the mutant T7-A-(VP2-K411R) into BSRT7, the T7 RNA polymerase stably expressing cell line, for 72 h. T7-B was transfected alone as a negative control and cotransfected with T7-A as a positive control. The cells were freeze-thawed three times and then centrifuged. The supernatant was incubated with the fresh DF-1 cells and cultured for 48 h, and cytopathic effect (CPE) was observed by a phase-contrast microscope.

### One-step growth curve.

DF-1 cells (KD, overexpression, or wild-type cells) were infected with WT or K411R IBDV at an MOI of 0.1. The cells were collected at the indicated time points. The infected samples were freeze-thawed three times and then centrifuged to collect the supernatant fraction. The 50% tissue culture infective dose (TCID_50_) was analyzed to determine the virus titers. Briefly, the supernatant fraction was diluted 10-fold to the dilution of 1 × 10^−10^ using DMEM containing 2% FBS, and then the diluted samples were used to infect fresh DF-1 cells. CPEs were recorded as positive samples.

### Statistical analysis.

Each experiment was repeated three times. Statistical difference was determined using Student's *t* test (***, *P* < 0.001; **, *P* < 0.01; *, *P* < 0.05; not significant [ns], *P* > 0.05). The results of the analyses are presented as means ± standard deviations (SD).
